# Rise of the Molecular Machines

**DOI:** 10.1002/anie.201503375

**Published:** 2015-07-27

**Authors:** Euan R Kay, David A Leigh

**Affiliations:** EaStCHEM School of Chemistry, University of St Andrews North Haugh, St Andrews KY16 9ST (UK) http://kaylab.wp.st-andrews.ac.uk; School of Chemistry, University of Manchester Oxford Road, Manchester M13 9PL (UK) E-mail: david.leigh@manchester.ac.uk Homepage: http://www.catenane.net

**Keywords:** molecular devices, molecular machines, molecular motors, molecular nanotechnology

## Introduction

When we get to the very, very small world … we have a lot of new things that would happen that represent completely new opportunities for design … At the atomic level we have new kinds of forces and new kinds of possibilities, new kinds of effects. The problem of manufacture and reproduction of materials will be quite different … inspired by biological phenomena in which chemical forces are used in a repetitious fashion to produce all kinds of weird effects (one of which is the author) … Richard P. Feynman (1959)^[2]^

It has long been appreciated that molecular motors and machines are central to almost every biological process. The harvesting of energy from the sun, the storing of energy, transporting cargoes around the cell, the movement of cells, generation of force (at both the molecular and macroscopic levels), replication, transcription, translation, synthesis, driving chemical systems away from equilibrium, etc.—virtually every biological task involves molecular machines.[1] Given the success of mankind’s machines in the macroscopic world, from the Stone-Age wheel to the modern-day smart phone, it was inevitable that we should one day seek to achieve the ultimate in machine miniaturization. However, it has taken some time to gain sufficient mastery over the necessary synthetic and supramolecular chemistry (and related physics) for this field to begin to flourish.

**Figure 1:**
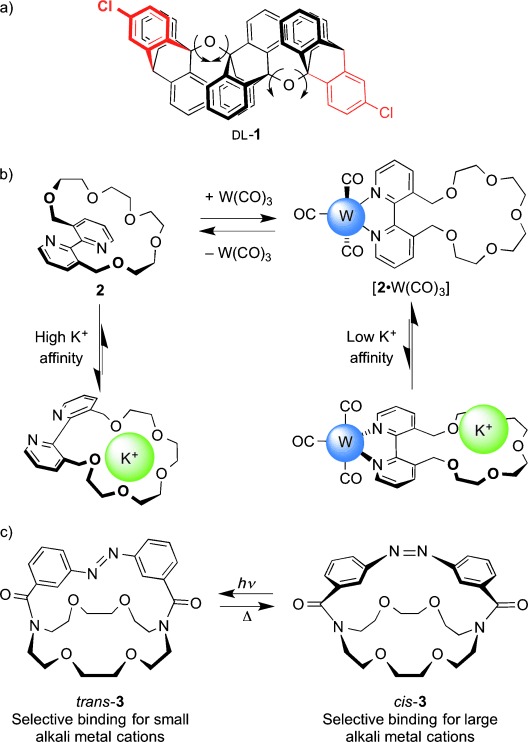
Correlated intramolecular motions within “proto-molecular machines”: a) Intramolecular mechanical cog-wheeling (this example, Iwamura et al.; 1983);[7] b) a negative heterotopic allosteric receptor (Rebek et al.; 1979);[8] c) photoswitchable binding of a crown ether (Shinkai et al.; 1980).[9]

Richard Feynman’s classic 1959 lecture *There’s plenty of room at the bottom*[2] outlined some of the promise that man-made molecular machines might hold, a scientific “taster” that Eric Drexler embraced for his controversial[3] vision of “nanobots” and “molecular assemblers”.[4] However, whilst inspirational in general terms, it is doubtful whether either of these manifestos had much practical influence on the development of artificial molecular machines.[5] Feynman’s talk came at a time before chemists had the synthetic methods and analytical tools available to be able to consider how to make molecular machines; Drexler’s somewhat nonchemical view of atomic construction is not shared by the majority of experimentalists working in this area. In fact, “mechanical” movement within molecules has been part of chemistry since conformational analysis became established in the 1950s.[6] As well as being central to advancing the structural analysis of complex molecules, this was instrumental in chemists beginning to consider dynamics as an intrinsic aspect of molecular structure and hence a property they could aspire to control. Artificial systems were designed to exhibit particular conformational behavior, such as the “cog-wheeling”-correlated motions of aromatic “blades” in triptycenes and related structures constructed by the groups of Ōki, Mislow, and Iwamura in the 1970s and 1980s (e.g. **1**, Figure 1 a).[7] Before long, stimuli-induced changes in conformation had been used to control molecular recognition properties; two of the seminal examples being Rebek’s use of allostery[8] (binding at one site influencing binding affinity at a second site; **2**, Figure 1 b) and Shinkai’s azobenzene photoswitch[9] for modulating the cation-binding properties of crown ethers (**3**, Figure 1 c). However, the field of synthetic molecular machines really began to take off with developments that occurred in the early 1990s.

## Architectures for Well-Defined Large-Amplitude Molecular-Level Motions

**Figure 2:**
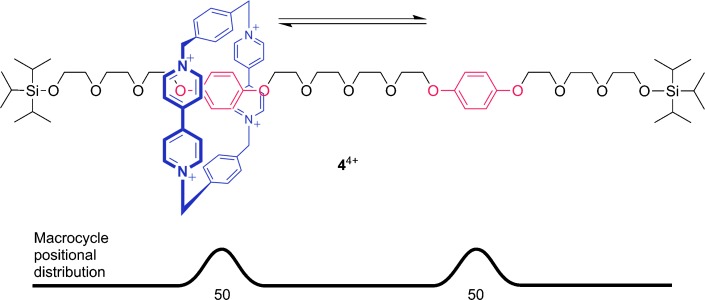
The first molecular shuttle (Stoddart and co-workers; 1991).[10]

In many ways the field of artificial molecular machinery began with J. Fraser Stoddart’s invention of a “molecular shuttle” (**4**) in 1991 (Figure 2).[10] In this rotaxane (a molecule with a ring mechanically locked onto an axle by bulky stoppers), the ring (shown in blue) moves between two preferred binding sites (the two hydroquinone units, shown in red) by random thermal motion (Brownian motion). The use of template effects to assemble mechanically linked molecules (catenanes and, later, rotaxanes) had been introduced by Jean-Pierre Sauvage in the early 1980s;[11] Stoddart’s great insight was to recognize that the threaded (mechanically interlocked) architecture of a rotaxane could allow for the large-amplitude motion of molecular-level components in a well-defined and potentially controllable manner. The authors of the 1991 JACS paper noted: “Insofar as it becomes possible to control the movement of one molecular component with respect to the other in a [2]rotaxane, the technology for building ‘molecular machines’ will emerge.”[10]

This statement turned out to be highly prescient and the paper hugely influential. Although mechanically interlocked structures are not necessary to construct molecular machines (see below), they provided the first practical synthetic molecular architecture through which well-defined large-amplitude molecular-level motions could be selectively addressed, studied, and utilized.[1b–g] This gave an exciting and compelling reason to make rotaxanes and catenanes, and the area burgeoned from these molecules being academic curiosities in the 1960s (when catenanes and rotaxanes were first made by long and/or inefficient synthetic routes[12]) through to 1989 (when Stoddart and co-workers made their first catenane,[13] six years after Sauvage and co-workers revolutionized the strategy for their synthesis through the use of template methods[11]) to the mainstream area it is now,[14] with hundreds of groups active in this field from the mid-1990s onwards.

## Switching the Relative Positions of Molecular Components—From Molecules to Machines

**Figure 3:**
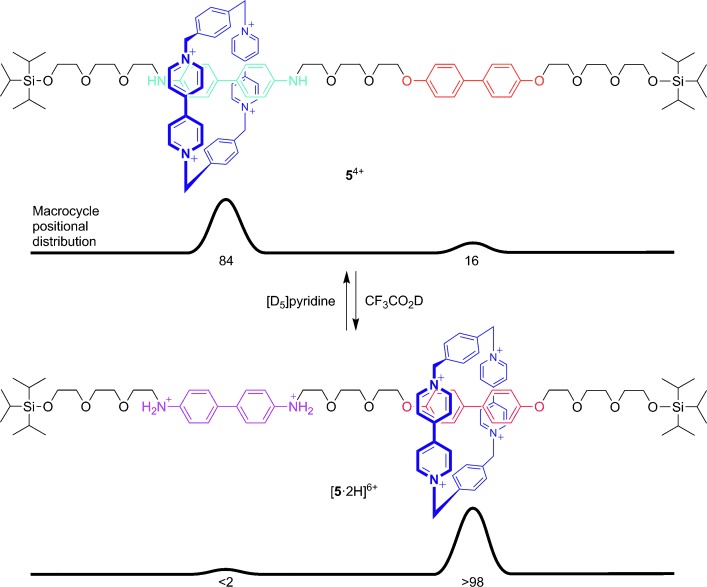
The first switchable molecular shuttle (Stoddart, Kaifer, and co-workers; 1994).[15]

By desymmetrizing a rotaxane thread to have two different potential binding sites, or “stations”, whose relative affinity for the ring could be switched, Stoddart, Kaifer, and co-workers arguably invented the first artificial molecular Brownian motion machine (**5**, Figure 3).[15] The cationic ring (shown in dark blue) prefers to reside over the benzidine group (shown in light blue) rather than the biphenol site (shown in orange). However, protonation (or electrochemical oxidation) of the benzidine station (now in purple) renders the biphenol group the preferred binding site for the cationic ring, thereby causing a net displacement of the ring along the track. This system marks the first example of a large-amplitude, well-defined, controlled switching of the position of a component along a molecular track.

*Euan Kay received his MChem from the University of Edinburgh in 2002 and his PhD in 2006 under the supervision of David Leigh. He was the recipient of a 2007 IUPAC Prize for Young Chemists. Following postdoctoral work in Edinburgh,* he *joined Prof. Moungi Bawendi at MIT (2008–2010). Since 2011*, *he has been a Royal Society of Edinburgh/Scottish Government Personal Research Fellow at the University of St Andrews. His research interests focus on translating dynamic and stimuli-responsive (supra)molecular systems into the nanoworld.*

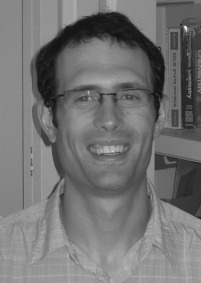

*David Leigh was born in Birmingham and obtained his BSc and PhD from the University of Sheffield. After postdoctoral research in Ottawa (1987–1989), he was appointed to a Lectureship at the University of Manchester Institute of Science and Technology. After spells at the Universities of Warwick and Edinburgh he returned to Manchester in 2012*, *where he currently holds the Sir Samuel Hall Chair of Chemistry. He was elected a Fellow of the Royal Society in 2009. His research interests include chemical topology and synthetic molecular-level motors and machines.*

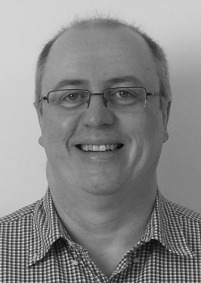

The groups of Stoddart and others (notably those of Sauvage, Balzani, Fujita, Hunter, Vögtle, Sanders, Beer, and Leigh) contributed to the development of many other rotaxane- and catenane-forming motifs and strategies over the period 1992–2007,[1b,f,g] and invented many different ways of switching the positions of the components in rotaxane and catenane architectures with various stimuli (light, electrochemistry, pH value, polarity of the environment, cation binding, anion binding, allosteric effects, temperature, reversible covalent-bond formation, etc).[1b,f,g] The next key advance needed was—and in some respects still is—to find ways to use the change of the position of the components of a molecular machine to perform useful tasks (see below).

### The Invention of Rotary Molecular Motors

**Figure 4:**
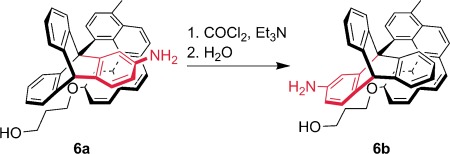
Chemically powered 120° directional rotation of a triptycene residue (Kelly et al.; 1999).[16]

In 1999, two papers appeared back-to-back on the subject of controlling the direction of rotary motion.[16, 17] The group of T. Ross Kelly used chemical reactions—urethane formation followed by hydrolysis—to bias a 120° rotation of a triptycene residue in one direction (**6**, Figure 4).[16] Unfortunately, Kelly was never able to extend this approach to a system where 360° rotation occurs directionally.

**Figure 5:**
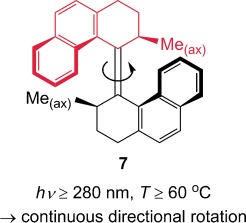
The first light-powered rotary molecular motor (Feringa and co-workers; 1999).[17]

The paper that followed it in the same issue of *Nature*, however, described an over-crowded alkene molecule (**7**) in which the components, upon irradiation with light, rotate directionally all the way around the alkene axis.[17] This molecule, from the Feringa group, was the first example of a synthetic rotary molecular motor and, indeed, the first example of an artificial molecular motor of any kind (Figure 5). Not only does this elegant design—which exploits, alternately, photoisomerization followed by a strain-induced diasteromeric helix inversion—achieve complete 360° rotation of one half of the molecule with respect to the other, it does so continuously as long as the compound is irradiated with photons and is above a critical temperature.

**Figure 6:**
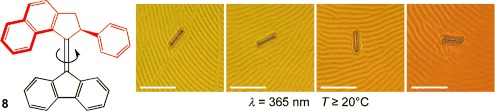
Rotating a macroscopic object with a molecular machine (Feringa and co-workers; 2006). Modified from Ref. [19] with permission.

**Figure 7:**
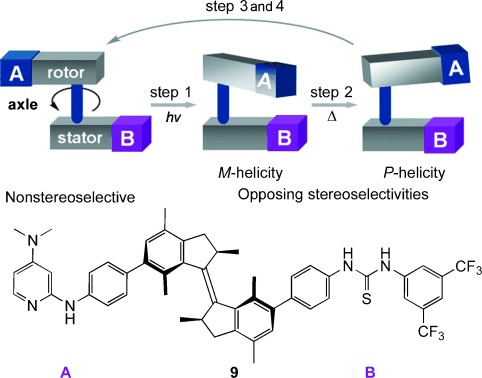
Changing the stereoselectivity of a nucleophilic organocatalyst with a molecular machine (Wang and Feringa; 2011). Modified from Ref. [20] with permission.

Over the next decade, many structural improvements were made to this type of motor molecule, dramatically increasing the rate of rotation[18] and using them to perform tasks, such as rotating a macroscopic object on the surface of a liquid-crystal medium (**8**, Figure 6),[19] switching the chirality of an organocatalyst (**9**, Figure 7),[20] and acting as the “motorized wheels” of a “nanocar”.[21]

### Brownian Ratchet Mechanisms

The problem of constructing a motor by using molecular components which move through random thermal motions distils down to achieving directional motion of Brownian particles. This issue has, at various times, intrigued some of the greatest physicists of the past 150 years: it is the essential process behind the celebrated thought experiments known as Maxwell’s demon (1871),[22] Smoluchowski’s trapdoor (1912),[23] and Feynman’s ratchet-and-pawl (1963)[24] (for a discussion of their relevance to artificial molecular machine design, see Ref. [1f]). In the last two decades of the 20th century, these superficially abstract deliberations gave way to a flourishing field of (mostly theoretical) studies that established a range of Brownian ratchet mechanisms through which directional motion of Brownian particles can be achieved.[25] These provide the mechanistic framework for the operation of all molecular motors—whether biological or artificial.[26] Unfortunately, however, chemists failed to appreciate these findings until the mid-2000s; most systems described as “motor-molecules” in the 1990s and 2000s were actually switches incapable of doing work cumulatively (i.e. any task performed is undone by the action of resetting the switch).[1f]

**Figure 8:**
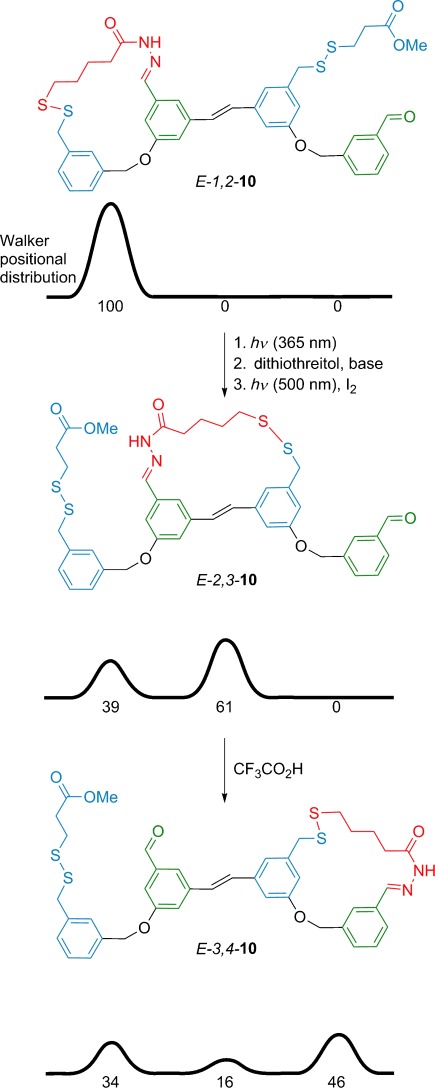
A small molecule that walks directionally along a molecular track using a light-fueled energy ratchet (flashing ratchet) mechanism (Leigh and co-workers; 2011).[30]

The first application of ratchet mechanisms to the de novo design of artificial molecular machines resulted in a catenane-based rotary motor[27] and was subsequently developed further over the next few years in a series of rotaxane-based machines that could pump macrocycles to higher-energy (non-equilibrium) distributions or states.[28] Yet, few synthetic linear small-molecule motors have been prepared to date. The first small molecule able to “walk” along tracks (reminiscent of the mode of transport of kinesin and other motor proteins) was reported in 2010,[29] with directional versions employing ratchet mechanisms introduced shortly afterwards (**10**, Figure 8).[30] Unlike rotaxane switches (e.g. **4**, Figure 2), these walkers move along their tracks progressively, each cycle in which fuel is consumed potentially causing the motor to take a step further along the track. Such devices are, in principle, capable of transporting cargoes directionally. However, current systems are only efficient enough to take a few steps along short tracks and are not yet sufficiently robust to walk across surfaces or along polymers.

A number of molecular walker–track systems that are either largely or entirely assembled from DNA building blocks have been described.[31] Many of these DNA walkers are genuine motors, since they exhibit all four of the fundamental characteristics of molecular motors: repetitive, progressive (that is, multiple operations of the motor do work cumulatively), processive (that is, take multiple steps without dissociating), and directionally biased transport of a molecular fragment (walker unit) along a track.[32] However, synthetic DNA walkers are generally of a similar size to, or even larger than, biological motor proteins such as kinesin-I, and their applications are likely to be more limited than that of wholly synthetic systems as they are restricted in terms of both operating conditions and chemical stability. Despite these limitations, the ease with which complex DNA constructs can be designed and made by automated synthesizers (often carried out to order by commercial suppliers), has meant that some enormous (250 000–500 000 Da) DNA-derived molecular machines have been prepared that can perform sophisticated tasks, such as transporting gold nanoparticles from place-to-place in a programmable “nano-assembly line”.[33] These systems benefit from the tremendous advances being made in other areas of DNA nanotechnology, such as DNA origami, DNA tiles, and DNA computing.[34]

## Design Philosophies: Should We Try To Mimic Biological or Macroscopic Machines?

Consider any machine—for example, an automobile—and ask about the problems of making an infinitesimal machine like it … Biology is not simply writing information; it is doing something about it. Richard P. Feynman (1959)

Over the last two decades, various types of molecular architectures have been used to make molecular “pistons”,[35] “clutches”,[36] “windmills”,[37] “elevators”,[38] “wheelbarrows”,[39] and even “nanocars”,[40] taking the appearance and modes of operation of macroscopic machines as their inspiration. However, just because a space-filling representation of a molecule looks like a macroscopic piston or automobile does not mean that the molecule can necessarily perform a similar function at the molecular level. Matter behaves very differently at different length scales, and random thermal motion, heat dissipation, solvation, momentum, inertia, gravity, etc. take on very different significances for the operating environment at the molecular level compared to what is important for macroscopic machine mechanisms.[1f,g, 41] Machines need to be designed according to the environment they are intended to operate in (for example, a car, intended for transport on solid ground, would not perform well either on water or in outer space!). However, mimicking biology is certainly not the only way to achieve complex functionality: computer chips are manufactured from silicon wafers rather than being wet and carbon-based like our brains. To date, it remains unproven as to whether making iconic molecular representations of macroscopic objects or following the principles of biological machines will be the most effective route for designing molecular machines with useful functions. Indeed, perhaps the most productive solutions will be found by following neither of these approaches too closely.

The tasks that molecular machines are best suited to carry out also need careful consideration:

“I can't see exactly what would happen, but I can hardly doubt that when we have some *control* of the arrangement of things on a small scale we will get an enormously greater range of possible properties that substances can have, and of different things that we can do.”

Richard P. Feynman (1959)[2]

### 1) Molecular Machines for Molecular Electronics

I can’t see exactly what would happen, but I can hardly doubt that when we have some *control* of the arrangement of things on a small scale we will get an enormously greater range of possible properties that substances can have, and of different things that we can do. Richard P. Feynman (1959).

**Figure 9:**
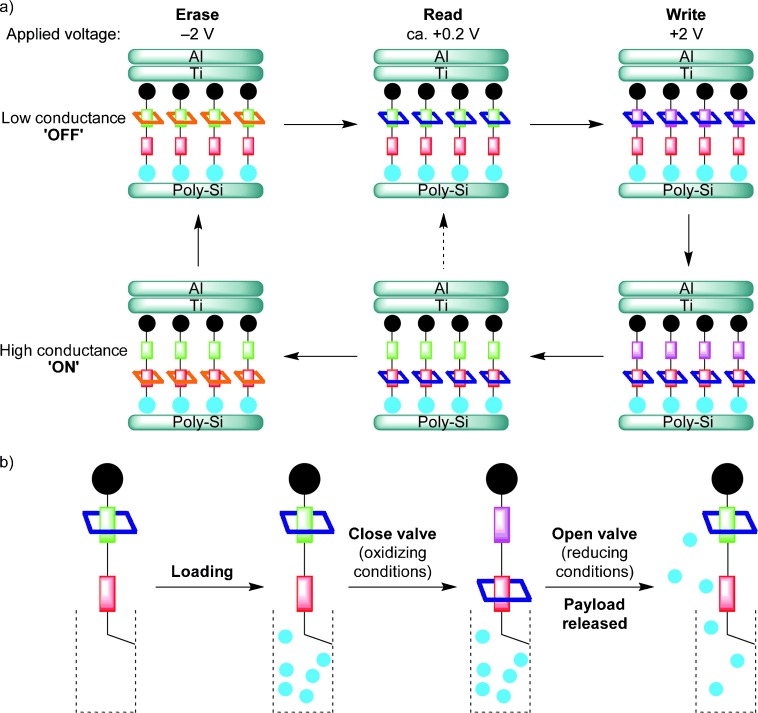
a) Rotaxane-based molecular switch tunnel junctions (Stoddart, Heath, and co-workers; 2007).[44] b) Controlled release of guest molecules using a rotaxane valve (Stoddart, Zink, and co-workers; 2005).[48]

In a series of controversial[42] and ground-breaking[43] experiments from about 1997 to 2007, the groups of Stoddart and Jim Heath interfaced switchable rotaxanes and catenanes with silicon-based electronics in an effort to try to use molecular shuttles in solid-state molecular electronic computing devices (Figure 9 a). Competing with the electronic movements in silicon and other semiconductors (which intrinsically occur billions of times faster than the change of position of the components in rotaxanes) seems somewhat counterintuitive as a problem suited for solving with abacus-like positional changes within molecular machines. However, a decade of effort and progress culminated in the fabrication and testing of a remarkable 160-kbit memory at 10^11^ bits cm^−2^ based on a monolayer of switchable rotaxanes as the data-storage elements.[44] Whether rotaxanes will ever be effectively employed in electronics remains an open question, but an important legacy of this pioneering research program is the vast amount learnt regarding how to interface complex functional molecules with silicon. These efforts also served to inspire the use of rotaxane-based switches to induce other types of macroscopically observable property changes through mechanical movement, including chiroptical switching (2003),[45] fluorescence switching (2004),[46] writing of information in polymer films (2005),[47] and in controlled-release delivery systems (Figure 9 b, 2005).[48]

### 2) Molecular Machines that Can Do Mechanical Work: “Molecular Muscles”

**Figure 10:**
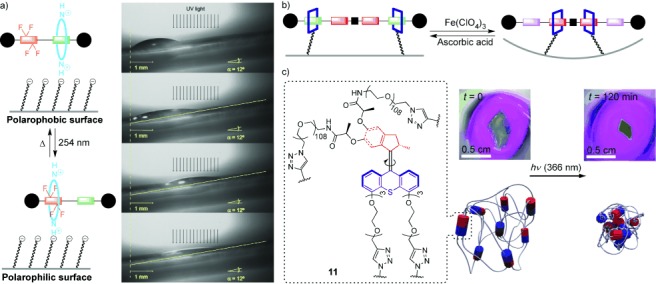
Artificial molecular machines put to work. a) A rotaxane molecular machine that does mechanical work by moving a liquid droplet against the force of gravity (Leigh and co-workers; 2005).[49] b) A rotaxane molecular machine that does mechanical work by bending a microcantilever (Stoddart and co-workers; 2005).[50] c) Transduction of molecular rotary motion into macroscopic contraction of a gel (Giuseppone and co-workers; 2015).[51] Parts (a) and (c) modified from Refs. [49] and [51] with permission.

Using controlled molecular-level motion to generate force in the macroscopic world is an appealing task for molecular machines because this is, of course, how muscles work. In 2005, the groups of Leigh and Stoddart each reported artificial molecular machines capable of doing mechanical work. Leigh’s group used the light-induced shuttling of a surface-bound rotaxane to mask a polarophobic fluorocarbon unit. The change in surface properties could be used to propel a droplet along a surface and up a slope, against the force of gravity (Figure 10 a).[49] Stoddart’s group used the contraction of a rotaxane as a molecular actuator to bend a gold microcantilever beam (Figure 10 b).[50] Recently, Giuseppone and co-workers described the use of light-driven molecular rotary motors to bring about macroscopic contraction of a gel (**11**, Figure 10 c).[51]

### 3) Molecular Machines that Can Make Molecules

Ultimately, we can do chemical synthesis. A chemist comes to us and says, ‘Look, I want a molecule that has the atoms arranged this and so; make me that molecule.’ Richard P. Feynman (1959)

**Figure 11:**
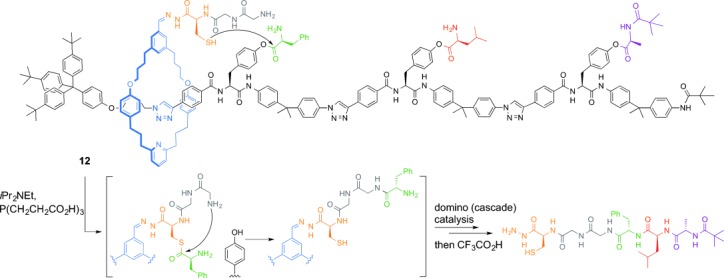
Making molecules with molecular machines: an artificial molecular peptide synthesizer (Leigh and co-workers; 2013).[52]

From polyketide synthase to DNA polymerases and the ribosome, one of the key uses of molecular machines in biology is for the construction of other molecules. In 2013 an artificial small-molecule machine was invented that assembled a tripeptide of specific sequence by travelling along a track loaded with amino acid building blocks (**12**, Figure 11).[52] This is a (very!) primitive analogue of the task performed by the ribosome in cells, but arguably one of the most sophisticated performed by an artificial molecular machine to date. For a synthetic molecular machine it has a truly complex mechanism of operation, requiring the integrated interaction of several functional component parts: a reversibly attached reactive “arm” with a regenerable catalytic site and a peptide-elongation site, a ring that threads the track catalytically with no residual ring–track interactions to retard the machine’s action, and a track with amino acid building blocks in a predetermined sequence separated by rigid spacers. Systems with integrated mechanisms of operations are likely to lead to increasingly ambitious and potentially useful applications of synthetic molecular machines.

## Outlook

‘Who should do this and why should they do it?’ Well, I pointed out a few of the economic applications, but I know that the reason that you would do it might be just for fun … … have some fun! Richard P. Feynman (1959)

The future for the field of artificial molecular machines appears very bright. There is already a working nanotechnology based on molecular machines that perform numerous useful tasks: it is called Biology. The natural world shows us just how exquisite and diverse the functions are that can be carried out with molecular machines. Advances in artificial systems over the past 25 years mean that chemists now have the know-how and synthetic tools available to enable them to make suitable machine architectures (e.g. catenanes, rotaxanes, over-crowded alkenes, molecules that walk upon tracks). They can switch the position of components (often by clever manipulation of noncovalent interactions between the various parts), they understand how to use ratchet mechanisms to create motor mechanisms, and are learning how to introduce them into more complex molecular machine systems.

However, there are still basic challenges to overcome. In contrast to motor proteins, powered by ATP hydrolysis or proton gradients, there are as yet no chemically driven synthetic small-molecule motors that can operate autonomously (i.e. as long as a chemical fuel is present), the closest counter-examples being the over-crowded alkene motors designed by Feringa and co-workers that rotate continuously under irradiation with light. Furthermore, although there have been some notable successes in using artificial molecular machines to bring about property changes, few have been shown to perform useful tasks that cannot be accomplished by conventional chemical means. This contrasts with the essential roles played by biological machines in numerous cellular processes. When this last step happens—and the rapid advances of the last few years suggest that that time is not too far away—then artificial molecular machines will start to become the extraordinary nanotechnology that Feynman predicted. Making that happen, as he suggested,[2] will doubtless be fun!
